# *The ‘Cultured’ Cow*: Analyzing the Role of the Cow’s Acclaimed Holiness in Indians’ Dairy Consumption Intentions

**DOI:** 10.3390/ani16050769

**Published:** 2026-03-01

**Authors:** Chirantana Mathkari

**Affiliations:** Department of Animal Behavior, Ecology, and Conservation, & Anthrozoology, Canisius University, Buffalo, NY 14208, USA; mathkarc@canisius.edu; Tel.: +1-716-888-2775

**Keywords:** human–animal relations, animal-based food, milk and milk products, religious beliefs, animal culturalization, bovine, bovine welfare

## Abstract

India, the world’s largest producer and consumer of milk, deifies cows. Contemporary Hindu religious beliefs bestow upon the cow the status of a mother who provides humans with life-sustaining food—milk. However, the role of this culturally shaped human–animal dynamic in Indians’ routine dairy consumption remains largely unknown. This study aims to understand the role of cow-related religious beliefs in Indians’ intentions to consume cow dairy products using the theory of planned behavior (TPB) model. A quantitative survey was conducted involving 559 Indian adults, utilizing a snowball sampling method. Employing structural equation modeling, the findings indicated that Indians’ dairy consumption intentions are affected by their attitudes, subjective norms, and perceived behavioral control towards the dairy products (*p* < 0.001). Subjective norms had the most notable influence on dairy consumption intentions (β= 0.29, *p* < 0.001), and cow-related religious beliefs were a significant moderator of this link (Δβ= 0.11, *p* < 0.01). These findings show that consuming cow dairy products is a religiously shaped social practice in India. They reveal a conceptual and physical ‘culturalization’ of the cow in Indian society through which the animal is simultaneously sacralized *and* commodified. This highlights a paradoxical situation where the demand for cow dairy products, which arises significantly from the cow’s sacred, mother-like status, in turn perpetuates the growth and sustenance of the same dairy industry that compromises her wellbeing (Mother-Milk paradox). This irony, therefore, challenges the assumptions surrounding the use of cow dairy products as a normalized socioreligious practice in India, questions the abuse of the cow’s acclaimed sacrality for capitalistic purposes, and calls for further research on Indians’ awareness of the cow’s animality and of the implications of the cow’s religious commodification on the animal’s wellbeing. In this way, a deeper appreciation of the role of sociocultural dynamics in human–animal relations can be obtained, and generate culturally sustainable human–bovine relationships which promote both human and animal wellbeing.

## Simple Summary

India, the world’s largest producer and consumer of milk, deifies cows. Contemporary Hindu religious beliefs bestow upon the cow the status of a mother who provides humans with life-sustaining food—milk. However, the role of this culturally shaped human–animal dynamic in Indians’ routine dairy consumption remains largely unknown. This study aims to understand the role of cow-related religious beliefs in Indians’ intentions to consume cow dairy products using an established psychological model. Upon surveying 559 Indian adults, the results showed that Indians’ intentions to consume dairy products were influenced by how the people around them perceived those products, and this influence was significantly more pronounced among Indians who considered the cow sacred. These findings indicate that consuming cow dairy products is a religiously shaped social practice in India. They reveal a ‘culturalization’ of the cow in Indian society through which the animal is simultaneously sacralized *and* commodified. This highlights a paradoxical situation where the demand for cow dairy products, which arises significantly from the cow’s sacred, mother-like status, in turn perpetuates the growth and sustenance of the same dairy industry that compromises her wellbeing (Mother-Milk paradox). This study highlights the largely overlooked but crucial role of sociocultural dynamics in human-animal relations and calls for the development of culturally sustainable solutions that promote both human and animal wellbeing.

## 1. Introduction

Animal-sourced and animal-based foods make up a significant component of food consumed worldwide [1,2,3,4]. The consumption of these foods varies from one community to another and is often determined by the availability of the food [2,5,6,7,8] and the sociocultural perspectives surrounding the animal product [7,9,10,11,12]. Sociocultural perspectives have the potential to shape the consumption of a food product by influencing its demand and thereby its availability, making the role of these perspectives in food consumption intentions worth inspecting. The religious beliefs that shape a community’s perspectives towards various farm animals, including pigs, sheep, goats, and cattle, can directly impact the consumption of the products derived from these animals [13,14,15,16,17,18]. However, the role of human–animal dynamics in the societal choices of animal products obtained from that animal remain fairly unexplored in the context of dairy consumption in the predominantly Hindu state of India.

India is the world’s largest producer and consumer of milk [19,20], with cows (*Bos taurus* and *Bos indicus*) and water buffaloes (*Bubalus bubalis*) (the latter henceforth referred to just as buffaloes) producing 97% of the total milk [21]. Milk and milk products contribute 25% of the nation’s total agricultural gross domestic product [22,23] and dairy farming provides two-thirds of the gross income of rural households [24], making the dairy industry a crucial component of the nation’s economy. In 2024, India produced 99.5 million metric tons of milk (18.02% of the milk produced worldwide) and consumed almost all of it (89 million metric tons), making India’s consumption four and a half times higher than the U.S. and three and a half times higher than the European Union [25]. With an annual consumption growth rate of 4.2% since 2000, the nation’s demand for milk and milk products has significantly risen over the past two and a half decades and is expected to continue rising in the coming years [19,25].

Contemporary Hindu religious beliefs in the largely Hindu nation of India regard the cow as holy, although the sacrality of the cow is not supported by any of the Hindu scriptures [26]. This acclaimed sacrality of the cow appears to exert an influence on the nation’s consumption of two cow-derived foods, namely beef and milk. Slaughtering the cow is considered sinful in current Hindu beliefs [15,26,27] and is therefore banned in the majority of Indian states [28,29,30]. This has resulted in a significantly low consumption of beef in the nation [31,32,33,34,35]. The cow-revering religious beliefs bestow upon the animal the status of a mother who provides humans with life-sustaining food—milk [14,36,37]. Resultantly, although the detrimental effects of Indian dairy practices on cow welfare are comparable to raising and slaughtering the cow for beef [38,39,40], current religious beliefs promote the consumption of cow milk and milk products as a way of respecting the cow’s sacrality [41,42,43]. Contrary to this cultural view of the cow, the other dairy animal of India, the buffalo, though associated with religious worth in prominent Hindu scriptures [44], is not currently considered holy and indeed goes completely unacknowledged in current Hindu culture [45]. The effects of this preferential veneration of the dairy animals on the dairy consumption of the world’s largest milk consumer are therefore worth exploring.

Against this background, I aim to investigate the mechanism by which the theory of planned behavior (TPB), developed to predict the behavior of consumers, reciprocally combines with cow-related religious beliefs to influence Indians’ intentions to consume cow milk and milk products.

## 2. Conceptual Framework and Hypotheses Development

**Figure 1:**
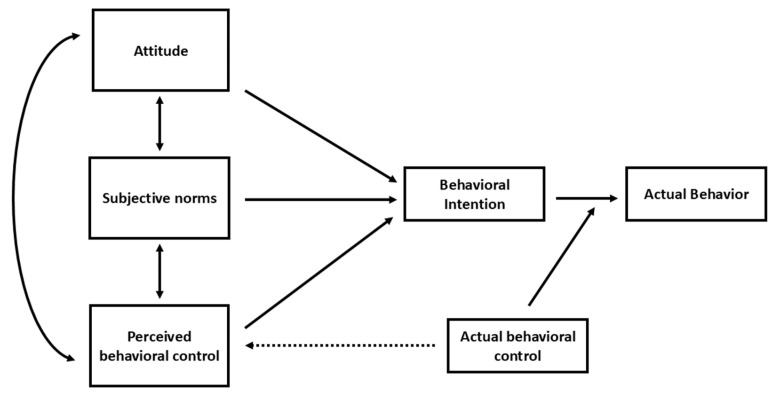
The theory of planned behavior (https://people.umass.edu/aizen/tpb.diag.html, accessed on 11 March 2025).

The theory of planned behavior (TPB), proposed by Ajzen [46,47], has been developed and applied extensively to better understand and predict human decision-making behaviors [48,49,50,51,52]. The theory, as illustrated in Figure 1, is based on the interactions of five key variables, namely: attitude, subjective norms, perceived behavioral control, behavioral intentions, and actual behavior. The perspectives of a human towards a specific behavior (attitude), the influence of others regarding that behavior (subjective norms), and the individual’s perceptions of self-efficacy and controllability of performing the behavior (perceived behavioral control) individually influence each other, and jointly impact the person’s behavioral intentions, which ultimately affect their actual behavior. The more favorable the attitude and the subjective norms towards performing the behavior, and the greater the perceived behavioral control, the more likely the individual is to form the behavioral intentions to perform the actual behavior.

### 2.1. TPB in Predicting Milk Consumption

Attitudes are often the strongest predictor of food consumption intentions [53,54] and have been seen to impact dairy consumption choices in India [55,56] and other nations [57,58,59,60,61,62,63]. Attitudes towards dairy consumption are determined by physio-affective and psychosocial processes [64,65,66,67], both of which appear relevant in the Indian dairy consumption scenario. The proportion of fat, a macronutrient in cow milk, significantly influences the taste of the milk [68,69,70], and thereby physio-affectively shapes attitudes towards its consumption [71,72,73,74,75]. This has been recently observed in the Indian context, where satisfaction levels were noted as a significant determinant of the attitude towards cow milk consumption [76]. Therefore, the commonly consumed milk in India, whole milk [77,78,79], which has a high fat content, is likely to create and sustain a positive attitude towards the consumption of this milk and the derived products. The social environment around dairy consumption in India, which involves the widespread visibility of cow milk and milk products in restaurants and retail shops [80,81,82,83] and ubiquitous dairy advertising messages and health-promoting campaigns [84,85,86], can influence the social experiences related to dairy consumption. Availability of the dairy product has been repeatedly shown to influence the Indian consumer’s attitude towards purchasing the product [87,88,89]. Additionally, Indians’ attitudes toward cow dairy consumption are often determined by the perceived health benefits of the dairy product [87,88,89,90]. This psychosocial process can therefore positively impact the attitudes towards dairy consumption in Indians. Hence, considering the sentiment-determining physiological and social contexts of dairy consumption in India, attitudes towards cow milk and milk products can reasonably impact Indians’ intentions to consume them.

Subjective norms affect food consumption in general [53,54,91], and dairy consumption in particular [55,92,93,94,95,96,97]. In the Indian context, this was recently seen where, among the three TPB constructs, subjective norms exerted the strongest influence on women’s dairy consumption intentions [55]. Informal as well as formal channels shape the subjective norms around dairy consumption [85,86,88,91], and both channels appear to play a role in Indians’ dairy consumption intentions. Close relations such as family and friends exert an impact on these subjective norms, with spouse and friends being a common influence on adults [60,98,99] and parents and siblings being influential on children [61,74]. India’s collectivistic family structure, where extended families and close-knit relationships are commonplace [100,101,102], can therefore be a significant informal influence on the individuals’ subjective norms. For example, in a recent Indian study on patterns of dairy consumption, family habits stood out as a significant determinant of norms around cow dairy consumption intentions [89]. Formal, health-related relations such as those with doctors, nurses, and nutritionists influence subjective norms regarding cow milk consumption [93,95,99]. Indian health professionals often endorse dairy consumption per the guidelines of the Indian Council of Medical Research, the nation’s apex medical research body [85,103,104,105,106], and have long been regarded with respect by Indians [107,108,109]. Trustworthiness has been repeatedly seen as an important influencer on Indians’ decision to consume cow dairy products [90,110]. This, therefore, makes Indian health professionals a potentially significant formal influencer on subjective norms. Thus, subjective norms, formed through formal and informal channels, can play a significant role in Indians’ dairy consumption intentions.

Perceived behavioral control is seen to influence dairy consumption by impacting self-efficacy and controllability, which the individual experiences when making decisions about buying, storing, and consuming milk and milk products [58,92,93,94,96,111]. Specifically in India, perceived behavioral control has been noted to have a significant impact on Indians’ dairy consumption intentions [55,56]. Multiple internal and external factors play a role in the perceived behavioral control of a behavior, such that an increase in one type causes a boost in the other [112]. Willingness and tolerability are both internal influencers on perceived behavioral control [92,112]. These can be significant in Indians given the staple nature of cow milk and certain milk products derived from the cow [84,113,114,115]. Other internal influencers of perceived behavioral control, such as affordability and time availability to purchase dairy [58,92,93,96], are applicable in the Indian scenario, considering the ubiquitous presence of dairy shops that provide easy access to largely affordable dairy products [82,115,116]. In fact, both price and convenience have been seen to play a role in shaping Indians’ perspectives towards consuming cow dairy products [87,88,89]. The common availability of dairy products, backed up by a consistently high milk production [19,20,25], exhibits the potential to act as an external influence on the perceived behavioral control of dairy consumption, as has been seen in other cultures [58,92,93]. Hence, the perceived behavioral control over dairy consumption, through internal and external factors, can reasonably impact dairy consumption intentions in Indians.

Based on this review of the TPB model, I posit the following hypotheses:

**H1**: *Indians’ attitudes regarding the consumption of cow milk and milk products will directly affect their intentions to consume cow milk and milk products.*

**H2**: *Indians’ subjective norms regarding the consumption of cow milk and milk products will directly impact their intentions to consume cow milk and milk products.*

**H3**: *Indians’ perceived behavioral control regarding the consumption of cow milk and milk products will directly impact their intentions to consume cow milk and milk products.*

### 2.2. Religious Beliefs as a Moderator

Religious beliefs, through shaping the individual’s environment, influence their values and norms, and therefore their behavioral patterns [117,118,119,120]. In the case of India, where the majority of the individuals adhere to religious beliefs [121] and where almost 80% of the total population identifies as Hindu [122], the influence of cow-related religious beliefs on Indians’ behavior warrants a scholarly investigation.

The ancient Hindu scriptures authenticate the customary slaughter of cows for food, medical treatment, domestic rituals, and religious sacrifices [26,123,124]. Although, over the centuries, a steady shift in the abandonment of cow slaughter occurred in Hindu religious practices in certain parts of India [26,125], regarding the cow as a holy, mother-like figure is not supported through any of the Hindu scriptures before, during, or after this shift [26]. Through the latter 1900s, the cow’s acclaimed sacrality was used to strategically flourish the newly independent nation’s dairy industry [84,126]. Socially ‘protecting’ the sacred cow was used as a political tool to attract Hindu votes immediately post-independence, and strongly resurfaced circa 2014 under the right-wing government of the nation [28,127,128]. Therefore, for Indians, the sacredness of the cow currently stands as a religious belief that requires continuous validation through external, authoritative sources rather than being an established truth of the Hindu religion [129]. Ironically, buffalo, the other dairy animal of India, although linked with religious significance in Hindu scriptures [44], is not regarded as holy in contemporary Hindu beliefs and goes completely unacknowledged in the nation’s current culture [45]. This contemporary acclaimed sanctity of the cow and the neglect of the religious worth of the buffalo is thus contradictory to the very ideology of Hinduism.

The acclaimed holiness of the cow has impacted Indians’ food choices in general and the consumption of cow dairy products in particular. Creation and sustenance of multiple societal boundaries, including cultural, economic, biological, political, and national discrimination, has occurred based on religiously shaped food choices and availability [130,131,132,133]. Food is also used in India to signal societal status, which is often measured through the quantity and quality of the food shared with community members. The quality of this food is often judged based on the inclusion/exclusion of cow dairy products in it [27,66,134]. The contemporary religious regard of the cow has led to a negative association with the consumption of beef and beef products in the nation. Although beef is only a by-product of the Indian dairy industry, and although culling the cow can compromise the animal’s wellbeing equivalent to that of abandoning the animal post-production [38,40,135,136], the consumption of beef is avoided by religious Hindus [26,124,137]. The deification of the cow has created a highly positive connotation towards her milk. The cow’s sacred mother-like status has led to the consideration of milk as life-sustaining food, which is provided to the Hindus by their ‘cow mother’ [40]. Cow’s milk is regarded as the core of birthing, purifying, and sustaining the order of the universe [27,66]. Therefore, its use has been linked to respecting the cow’s sacrality and to gaining and maintaining ritual purity [41,64]. This contemporary religious belief has led to a significant cultural regard for cow’s milk and milk products, resulting in their widespread use in religious rituals. Cow dairy products, such as clarified butter, condensed milk, fermented milk, and cream, are used as a base for preparing festive foods, while a mixture of other cow dairy products, such as yogurt, milk, and clarified butter, along with honey and sugar, is used as an offering to the Hindu deities which is then distributed among the devotees for consumption [27,138]. However, how the religious beliefs surrounding the cow impact Indians’ everyday consumption of cow milk and milk products remains largely unknown.

Based on this review of the literature, I therefore ask the following questions:

**RQ1**: Do cow-related religious beliefs moderate the effect of Indians’ attitudes towards the intention to consume cow milk and milk products?

**RQ2**: Do cow-related religious beliefs moderate the effect of Indians’ subjective norms towards the intention to consume cow milk and milk products?

**RQ3**: Do cow-related religious beliefs moderate the effect of Indians’ perceived behavioral control towards the intention to consume cow milk and milk products?

Although the role of cow-related religious beliefs in Indians’ routine cow dairy consumption has not been tested previously, based on the established influential role of religious beliefs in the TPB constructs of Indians’ food consumption [56,132,139,140,141,142], an amplifying moderation of religious beliefs is anticipated in this study.

**Figure 2:**
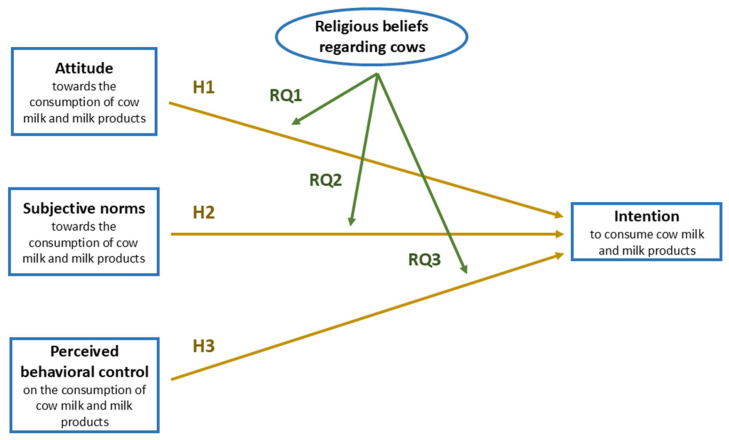
Proposed conceptual framework, based on the theory of planned behavior. Golden arrows represent routes exploring direct positive effects between the linked variables, while green arrows indicate the routes exploring the moderating effects. It is hypothesized that Indians’ attitudes (H1), subjective norms (H2), and perceived behavioral control (H3) regarding the consumption of cow milk and milk products will directly affect their intentions to consume cow milk and milk products. Through the research questions, I aim to understand how religious beliefs regarding cows influence the relationship between Indians’ attitudes and cow milk consumption intentions (RQ1), their subjective norms and cow milk consumption intentions (RQ2), and their perceived behavioral control and cow milk consumption intentions (RQ3).

The framework, hypotheses, and research questions of this study are illustrated in Figure 2. Expanding on the TPB model, the key variables identified for the framework of this study include attitude, subjective norms, perceived behavioral control, and religious beliefs regarding cows.

## 3. Materials and Methods

The research protocol for this study was approved by the Institutional Review Board of the University of Maryland, College Park, USA (IRB approval no.: 1669971-1).

### 3.1. Participants and Procedures

A total of 559 Indians participated in this study. The sample consisted of healthy Indians who had resided in India for at least 10 years, to minimize any cultural biases in the participants’ responses [143]. All participants were over the age of 18 years, with the majority being under 35 years of age (73%). More than 63% of the participants identified as females, 36% as males, and 0.5% as being non-binary. The majority of participants were educated with more than 36% holding a bachelor’s or a diploma degree, 42% holding a master’s degree, and 5% held doctorates. 85.7% of the respondents identified as Hindus, and other represented religions included Jainism (5%), Islam (4.1%), Buddhism (2.5%), and Christianity (1%). Although this religious representation largely reflects the situation where the majority of Indians identify as Hindus, note that the participants still may not be an accurate representative of the general population of India.

Informed consent forms, which explained the purpose and process of the research in detail, the participants’ rights to participate in the study, and the anonymity of their responses, were signed by the participants before completing the questionnaire. An online questionnaire was designed to assess Indians’ cow dairy consumption intentions in relation to the identified key variables of attitude, subjective norms, perceived behavioral control towards cow dairy consumption, and religious beliefs regarding cows. The survey was made available in three languages: the widely spoken official language, English [144,145,146], the second official language, Hindi (Article 343, Constitution of India 1950), and one regional language, Marathi, such that the participants could complete the questionnaire in the language of their choice. The original survey was designed in English. The researcher then translated the survey items into Hindi and Marathi. These translated surveys were then back-translated using Brislin’s method [147] by independent trilingual professionals to ensure accuracy. The researcher sent an invitation to participate in the online survey via social media and e-mail accounts (e.g., WhatsApp, Facebook, and Gmail). Using Goodman’s snowball sampling technique [148], the participants were asked to share the survey invite with their acquaintances who met the inclusion criteria of the study. The survey took about 13 min to complete. Data collection was carried out between November 2020 and April 2021.

### 3.2. Measures

The survey included 9 demographic questions and 12 questions related to attitude, subjective norms, perceived behavioral control towards cow dairy consumption, religious beliefs regarding cows, and intentions to consume cow milk and milk products. All measurement scales used in this study were adapted from well-established academic literature and were those that had been previously validated in existing research. The survey was administered online using a questionnaire, and the responses were recorded on a five-point Likert scale, with options ranging from ‘strongly disagree’ to ‘strongly agree’.

The survey items were based on Ajzen’s foundational study on the suitability of TPB in studying food consumption decisions [54] and were fortified using Ajzen’s TPB questionnaire construction guidelines [149]. This scale has been validated through multiple previous dairy consumption behavioral studies, which have adapted and used the scale [58,63,65,72,92,93,95,97,98,150,151], reflecting its extensive scholarly use and supporting its suitability for studying dairy consumption decisions. Intentions of dairy consumption were measured using two items focused on Indians’ anticipated likelihood and frequency of consuming cow milk and milk products. Three items each were utilized to capture Indians’ attitudes, subjective norms, and perceived behavioral control towards cow milk and milk product consumption, while religious beliefs regarding cows were measured using a single item that asked the participant to answer the following question: “Do you consider the cow sacred?”. Religious beliefs regarding cows are associated with multiple nuances, such as ceremonial observance and socioreligious identity, many of which can be culturally sensitive. The use of a single-item measure, which focuses objectively on the holiness of the cow, was therefore intentional to minimize any ethical burden on the participants [152,153]. The variables were measured using a five-point Likert scale that ranged from ‘1 = strongly disagree’ to ‘5 = strongly agree’. Confirmatory factor analysis (CFA) was used to measure the factorial validity of the key variables, and reliability tests (Cronbach’s alpha) were performed to ensure internal consistency of each multi-item construct. All the constructs showed an acceptable internal consistency, as reported in Table 1. Although the scale adapted in this study has been previously tested and validated in numerous studies in the specific context of dairy consumption decisions, an extensive psychometric validation was not performed before circulating the survey due to time constraints; therefore, a further psychometric analysis could strengthen the rigor of future research projects. The detailed survey items and format are presented in Table 1 to allow future replication.

### 3.3. Data Analysis

This study used SPSS 22.0 and AMOS 23.0 for statistically analyzing the data. The data was first sorted and analyzed in Microsoft Excel (Version 2601). SPSS was utilized for performing descriptive statistics, regression analyses, and reliability analyses, while AMOS was used for the confirmatory factor analysis (CFA) through structural equation modeling (SEM). SEM was selected due to its ability to assess the complex relationships between the variables, including the analysis of moderating and mediating effects. CFA helped examine the factorial validity of the key measures in the study (i.e., attitude, subjective norms, perceived behavioral control, and intention), while SEM was utilized to assess the sequence of the proposed associations within the TPB constructs and between the TPB constructs and religious beliefs. The participants’ age (average age 18–34 years old), gender (63.4% female, 36.01% male, 0.59% non-binary), and education, which was an ordinal variable measured by 10 categories (1 = no formal education; 10 = doctorate, M = 7.52, SD = 1.71) served as confounding factors and were controlled in the model.

A total of four goodness-of-fit indices (i.e., the root mean square error of approximation (RMSEA), the standardized root mean square residual (SRMR), the Tucker–Lewis index (TLI), and the comparative fit index (CFI)) were used to assess the overall fit of the proposed models [154]. Models were considered to have acceptable goodness of fit if the RMSEA and SRMR values were less than 0.08 and the TLI and CFI values were greater than 0.90 [155,156].

## 4. Results

Overall, the model explained 63.8% of the variation in intention (R^2^ = 0.638). The factorial validity of the model, measured through confirmatory factor analysis, was high (χ^2^/df = 2.85, RMSEA = 0.060, SRMR = 0.049, TLI = 0.913, CFI = 0.906). The proposed model yielded a strong fit to the data (χ^2^/df = 3.01, RMSEA = 0.058, SRMR = 0.041, TLI = 0.910, CFI = 0.904).

**Figure 3:**
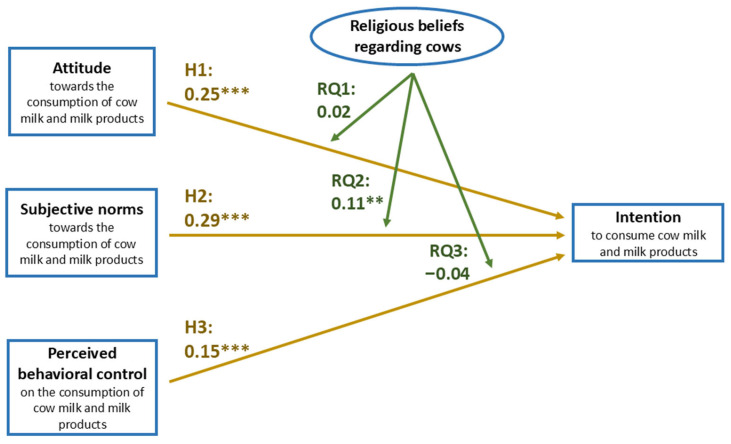
Structural model results of the regression analysis based on the theory of planned behavior, showing standardized path coefficients for factors influencing intentions to consume cow milk and milk products. Golden arrows represent routes of direct positive effects between the linked variables, while green arrows indicate the routes of the moderating effects. Attitude (β = 0.25, *p* < 0.001), subjective norms (β = 0.29, *p* < 0.001), and perceived behavioral control (β = 0.15, *p* < 0.001) were all positively associated with behavioral intentions, supporting hypotheses H1, H2, and H3, respectively. Addressing RQ2, the regression analysis further revealed that religious beliefs surrounding cows significantly moderated the influence of subjective norms on cow dairy consumption intentions (Δβ = 0.11, *p* < 0.01). ** = *p* < 0.01, *** = *p* < 0.001.

As illustrated in Figure 3, the analysis confirmed that the intentions to consume cow milk and milk products were positively associated with attitude (β = 0.25, *p* < 0.001), hence proving H1. Additionally, H2 was validated as the results revealed a positive association between behavioral intentions and subjective norms (β = 0.29, *p* < 0.001). Among the three TPB constructs, subjective norms exhibited the strongest association with behavioral intentions. Finally, perceived behavioral control and behavioral intentions exhibited a positive link (β = 0.15, *p* < 0.001), hence supporting H3.

Moderation by religious beliefs was examined using multi-group analysis. Before comparing structural paths across groups, measurement invariance was assessed to ensure that the constructs were interpreted equivalently across levels of religious beliefs. Configural invariance was first tested to establish that the overall factor structure was similar across groups. Metric invariance was then examined by constraining factor loadings to be equal across groups and comparing model fit to the configural model. As measurement invariance was supported, the sample was subsequently divided into groups based on levels of religious beliefs (strong and weak), and the structural model was estimated separately for each group. Differences in path coefficients across groups were assessed by comparing constrained and unconstrained structural models to determine whether the relationships significantly varied as a function of religious beliefs. A chi-square difference test indicated that constraining the subjective norms → intentions path to be equal across groups significantly worsened the model fit (Δχ^2^ = 5.28, Δdf = 1, *p* < 0.05), suggesting that religious beliefs moderate this relationship. Specifically, subjective norms were a stronger predictor of intentions in the participants who considered the cow sacred (β = 0.42, *p* < 0.01) than in those who did not (β = 0.31, *p* < 0.05). In answering RQ2, the researcher found that religious beliefs surrounding the cows exhibit a moderating effect in the association between subjective norms and cow dairy consumption intentions (Δβ = 0.11, *p* < 0.01). The data did not exhibit statistical significance in answering RQ1 and RQ3.

Cow milk consumption intentions were not influenced by the confounding variables of age (β = −0.02, *p* > 0.05), gender (β = −0.11, *p* > 0.05), and education (β = 0.07, *p* > 0.05) (ΔR^2^ = 0.140).

**Figure 4:**
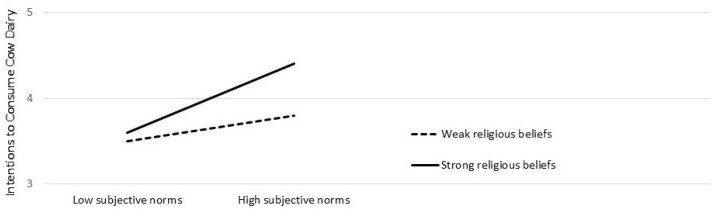
An interaction graph depicting the direction and magnitude of cow-related religious beliefs on the relation between subjective norms towards cow dairy products and the intentions to consume them.

An interaction graph was plotted to understand the moderating effect better. Figure 4 presents a detailed analysis of how religious beliefs influenced the relationship between subjective norms towards cow dairy products and intentions to consume them. High levels of cow-related religious beliefs enhanced the impact of subjective norms towards cow dairy products on the intention to consume the same. Essentially, participants with strong religious beliefs had more intention to consume cow dairy products, and more positive subjective norms towards cow dairy products strengthened this positive relationship.

## 5. Discussion

This quantitative non-experimental study investigated the role of cow-related religious beliefs in Indians’ intentions to consume cow dairy products through a structural model based on the theory of planned behavior. Employing an SEM analysis, this study found that Indians’ cow dairy consumption intentions are affected by their attitudes, subjective norms, and perceived behavioral control of dairy consumption, and that religious beliefs surrounding the cow play a significant role in moderating Indians’ subjective norms towards dairy consumption intentions.

This research responds to the call of scholars suggesting the need for more behavioral research to examine the TPB model in a non-Western context [157,158,159]. The significant impacts of attitudes, subjective norms, and perceived behavioral control on dairy consumption intentions seen in this study echo the results of previous dairy consumption studies performed in a Western context [60,92,95,160]. Therefore, the results not only reconfirm the importance of the TPB construct in examining food consumption patterns but also demonstrate its usefulness in studying animal-based food consumption intentions in an Indian context.

The influence of attitude on Indians’ cow dairy consumption intentions exhibits the nationals’ inherent positive outlook towards cow’s milk and milk products. This positive approach appears to stem from two distinct yet interconnected elements: pleasure and health. Deeming dairy as enjoyable indicates the presence of pleasure in consuming it, while considering dairy as beneficial suggests a health-based approach towards its consumption. Health-related behaviors that provide pleasure are more prone to sustaining and becoming a lifestyle than those that do not [161,162,163]. This relation, therefore, signifies the long-standing role of attitude in the creation of a habitual nature of cow dairy consumption in Indians.

Perceived behavioral control’s significant relationship with Indians’ cow dairy consumption intentions shows the role of autonomy in their dairy consumption. Indians have and seem to practice the choice of whether they want to consume cow dairy products and when they want to consume them. However, this autonomy appears not to be independent of their significant positive attitudes towards cow dairy consumption and the subjective norms surrounding the consumption of cow milk and milk products, making cow dairy consumption in India a result of socially influenced individual decisions. Access to cow dairy products is another determinant of Indians’ perceived behavioral control and is dependent on the products’ physical availability and affordability [164,165,166,167]. Therefore, Indians’ significant perceived control in consuming cow dairy products despite the continually rising cow dairy prices in the nation [115,168,169,170] is only a further indication of the habitual and socially informed nature of this behavior.

Subjective norms are the strongest determinant of Indians’ cow dairy consumption intentions, indicating the role of India’s collectivistic culture on the nationals’ cow dairy consumption. Indians’ cow dairy consumption intentions are driven largely by their community’s approach towards consuming cow dairy products. Both formal and informal relations form a part of this community and together shape the individual’s approach towards consuming the dairy products. Therefore, cow dairy consumption among Indians emerges as an act of conformity, stemming from strong family orientation, respect for certain community members (such as the elderly and doctors), and identifying with and wanting to be a part of the community. The cultural normalization of cow dairy consumption, accompanied by a lack of space for personal privacy in a collectivistic culture [171,172], only amplifies the intention to consume dairy products. This is achieved by minimizing the individual’s ability to rethink the norm and by perpetuating the fear of being ostracized for going against accepted sociocultural practice. Perception of cow dairy consumption by Indians who have resided or are currently residing abroad, in a different social environment, is therefore worth investigating.

In this study, Indians who regarded the cow as holy exhibited a notable influence of subjective norms on their cow dairy consumption intentions, signifying that the Indians who view the cow as a divine figure are more likely to consume cow dairy products as a social practice rather than a private behavior. This religious symbolization of the cow as a deity and as a maternal figure has caused a ‘culturalization’ of cow dairy products with the purpose of encouraging the consumption of these products by Indians. This culturalization is visible in the marketing strategies of multiple Indian dairy brands, which use words and images that highlight the religious affiliation of the cow’s milk or milk product. Words that exhibit the cow’s religious, maternal nature, such as *Mother Dairy*, *Nandini* (meaning ‘the divine cow’), and *Gokul* (meaning ‘the abode of cows’), are often used as brand names [173,174]. Words that denote the product’s association with the cow are used to label the product, and examples include ‘*desi* cow milk’ (*desi* meaning ‘native’), ‘*pure* cow ghee’ where pure denotes the nativeness of the cow and therefore the purity of the milk product derived from the animal, and ‘*A2* cow milk’ where the A2 stands for the A2 beta-casein, a distinctive protein found in the native cows’ milk [175,176,177,178]. Images that portray the cow in a religious light, such as those showing her as a decorated goddess or as standing alongside the cowherd and cow-protecting god *Krishna*, are not uncommon on the packages of Indian dairy products [37,169,172]. Therefore, Indians’ cow dairy consumption surfaces as a religiously influenced social practice that is born out of the cow’s cultural iconization and is reinforced through the emblematization of the animal’s milk and milk products. Studies researching how the traditionally adherent and observant populations of India, such as the rural, the elderly, and the less educated [179,180], perceive the consumption of cow dairy products can further our understanding of the role of cow-related religious beliefs in this context. Also worth exploring are potential gender differences in the perception of cow sacredness and cow dairy consumption, especially given the long-standing association of Indian women with religious compliance [181,182]. As compared to men, women are traditionally known to choose a vegetarian or a vegan lifestyle due to a more caring and nurturing approach towards animals [183,184,185,186]. However, in the current case, there appears to be a religious masking of the cow’s animality. This is only intensified by the suburban and rural locations of the Indian dairy farms [187] which create a curtain between the dairy consumer and the source of the dairy product. Therefore, Indian women might be more prone to consuming cow dairy products solely based on their stronger religious adherence than men.

The cow, then, in the world’s largest producer and consumer of milk, does not just remain a milk-producing farm animal, but rather becomes a ‘cultured’ entity. The Indian cow is cultured in two distinct yet interconnected ways. Firstly, she is cultured conceptually by inculcating her in the nation’s customs and traditions, consciously as a religious process and subconsciously as a routine process. Secondly, the cow is cultured physically by farming and therefore cultivating her species for obtaining milk. The physical culturing of the cow, which began a few decades after India’s independence from British rule, was performed as an attempt to uplift the nation financially through organizing its dairy sector [126]. However, in contemporary India, given the nation’s significant and ever-rising demand for dairy products [19,25], the physical culturing comes to light predominantly as a response to this consumer demand. This consumer demand, as visible in the results of this study, in turn emerges through the conceptual culturing of the cow. Therefore, the animal’s physical and conceptual culturing surface as two interdependent and intertwined processes. The de-individualization of the cow through her physical culturing and the cultural perception of the cow as a religious concept deprives Indians of the ability to view her as a singular, non-human animal. This obscuration of the cow’s animality plausibly prevents the nationals from perceiving the effects of the culturing on the dairy cow’s wellbeing.

**Figure 5:**
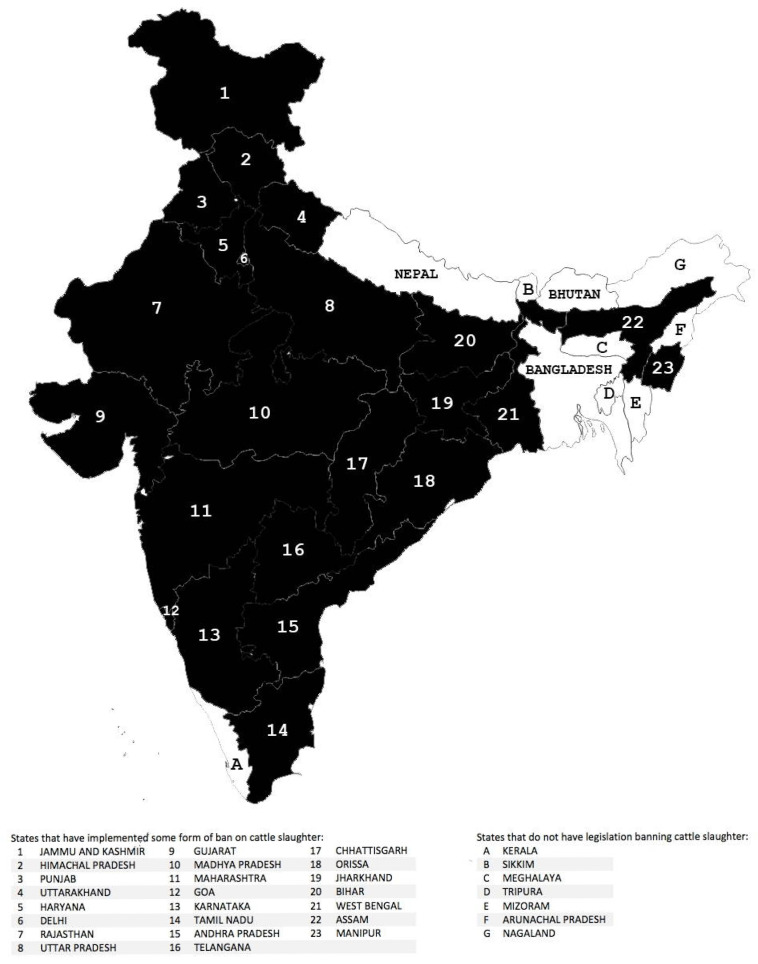
Political map of India with shaded areas indicating states that have implemented some form of ban on cattle slaughter. Reproduced from Parikh and Miller, 2019. *ACME: An International Journal for Critical Geographies*, 18(4), 835–874 [195], available under a Creative Commons Attribution 4.0 License.

The Indian dairy cow’s wellbeing stands compromised, with a loss of one or more of the Five Freedoms of animal welfare during her productive life and a parallel loss of two or more of the freedoms post-productivity [38]. Unlike several other nations, where a farm animal’s wellbeing is mainly determined by the farmer’s financial ability [188,189,190,191,192,193,194], the wellbeing of the Indian dairy cow is determined by three marked factors, which also exhibit interdependency: the sociocultural norms, the laws, and the finances surrounding the animal. An archetypal example of this interplay is the widespread cow slaughter ban in the nation (Figure 5) and its effects on the cow’s wellbeing. This cow slaughter prohibition directive, implemented soon after India’s independence from British rule, originated from and was used as a sociopolitical scheme to boost nationalism through utilizing the cow’s holy, Hindu mother identity to promote Hinduism and to attract Hindu votes [28]. This symbolic approach towards the cow, which stems from anthropocentric affective thought and perpetuates the deific and mother-like viewing of the animal, can limit a critical analysis of the effects of the slaughter ban on the cow’s wellbeing. The cow slaughter ban today prevents Indian dairy farmers from sending the majority of the unproductive cattle for slaughter, leading to their abandonment of the cows or the rearing of the unproductive animal until her death [38]. The latter is economically taxing for the farmers and limits the resources available to provide adequate feed, shelter, and veterinary care, thereby compromising the entire herd’s welfare. This legal binding especially negatively impacts the small-scale dairy farms of India, which make up the majority of the dairy farms in the nation [187]. It therefore warrants further nuanced research that analyzes the role of the regional (state and local) bovine-related laws in shaping the sale, purchase, and/or abandonment of the dairy cows, and in impacting the management practices surrounding the animals.

The current Hindu religious faith ironically impacts the wellbeing of both dairy cows and buffaloes negatively. On one hand, the religiously shaped cow slaughter ban ultimately causes the post-production abandonment of the animals on streets or in cow shelters, both being places where the animals’ wellbeing is compromised [38,195]. On the other hand, a thorough disregard of the buffalo’s historic religious significance [26,44] has caused the lack of any legal regulations around the animal, making buffaloes a disposable commodity whose wellbeing is not a fundamental concern for the farmer [45]. Therefore, Indians’ consumption of cow dairy products seems to arise more from the cultural idolization and maternalization of the cow rather than from their understanding of the animal’s treatment in the dairy industry.

**Figure 6:**
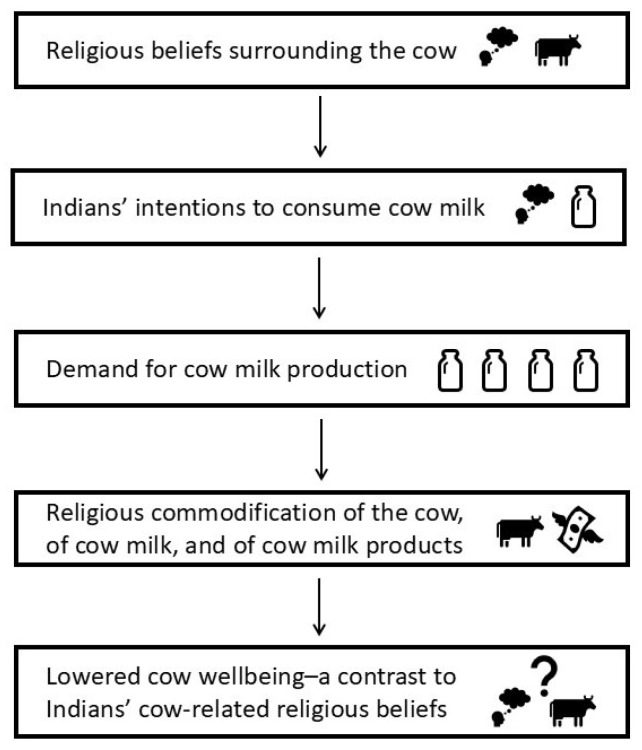
Visual representation of the Mother-Milk Paradox. This interpretive, conceptual framework hypothesizes the process through which the religiously influenced consumption of cow milk leads to a socioreligious commodification of the cow and her milk. This commodification results in legal obligations that prevent the selling of sub-productive or unproductive cows, creating a financial burden for ubiquitous small-scale farmers. The financial distress, in turn, jeopardizes the wellbeing of the entire cow herd, making the animal’s compromised wellbeing stand out as a stark contrast to the contemporary religious beliefs that idolize the cow mother.

This study highlights a paradoxical situation where the demand for cow dairy products, which arises significantly from the cow’s sacred, mother-like status, in turn perpetuates the growth and sustenance of the same dairy industry that compromises her wellbeing (Mother-Milk paradox) (Figure 6). This interpretive, conceptual framework, informed by the study’s results and the researcher’s prior scholarship [38], underlines the process through which the symbolization of the cow as a religious, sacrificing mother figure leads to the de-animalization of the bovine. It also causes a religious commodification of the cow, of her milk and of the milk products derived from this milk. This commodification, along with the sociocultural obscuration of the cow’s individuality, results in legal obligations that prevent the selling of sub-productive or unproductive cows, creating a financial burden for small-scale farmers. This financial distress, in turn, jeopardizes the wellbeing of the entire cow herd, making this compromised wellbeing stand out as a stark contrast to the contemporary religious beliefs that idolize the cow mother. The hypothesis of the Mother-Milk paradox, therefore, challenges the assumptions surrounding the use of cow dairy products as a normalized sociocultural practice in India, and questions the abuse of the cow’s acclaimed sacrality for capitalistic purposes. It calls for further research on Indians’ awareness of the cow’s animality and of the welfare implications of dairy farming on the animal, to obtain a deeper appreciation of the role of sociocultural dynamics in human–animal and human–food relations.

### Limitations of the Study

Despite the pioneering nature of this study in analyzing the role of cow-related sociocultural dynamics in Indians’ dairy consumption intentions, this study presents certain limitations. The study assessed Indians’ milk consumption intentions, but not actual behavior. As per TPB, past behavior exhibits a tendency to impact future intentions, and present intentions influence future behavior [47,51]. Therefore, the results for Indians’ milk consumption intentions can be considered an outcome of their previous milk consumption behavior and a predictor of their future milk consumption behavior. Still, if future research can track participants’ actual actions, it would help strengthen the analysis of this topic. Although the established constructs of the TPB were measured using multi-item validated scales, the religious beliefs regarding cows were measured using a single-item scale to maintain objectivity and observe ethical considerations. This was done as the deification and maternalization of the cow is a culturally sensitive topic. Although multiple psychometric assessments, such as CFA, Cronbach’s alpha, and four goodness-of-fit indices were utilized in the study, the use of more psychometric measures, including a test–retest reliability and a measurement equivalence, could have strengthened the study’s methodology. Therefore, future studies building and validating multi-item scales to ethically assess religious beliefs in general and the sacredness of the cow in particular can prove useful to study multiple human–cow and human–milk relationships. This study is limited by the use of snowball sampling and, therefore, the predominance of young, highly educated respondents. This urban and educated bias may limit our understanding of the effects of the cow’s sacredness on cow milk consumption by the more religiously conforming segments of India, such as the rural, the elderly, and the less educated populations [179,180]. The study survey was available in three different languages used in the nation, aiding Indians belonging to different educational levels and from both urban and rural settings across multiple states to participate in the study. However, future studies should focus on exploring the role of age, education, economic level, and sociogeographical settings in Indians’ approach towards cow milk consumption.

## 6. Conclusions and Future Directions

This empirical study sheds light on the role of cow-related religious beliefs on Indians’ cow dairy consumption intentions, specifically through the lens of the theory of planned behavior. By exploring the roles of religious beliefs, attitudes, subjective norms, and perceived behavioral control, this study confirms the significant influence of these variables on behavioral intention. Furthermore, religious beliefs are identified as a key moderating factor, particularly in strengthening the relationship between subjective norms and behavioral intention. The findings suggest that consuming cow milk and milk products is a religiously influenced social practice in India occurring in association with the deification and the mother-like symbolization of the cow, as well as the culturalization of her milk and milk products in the nation. This study offers both theoretical insights into this unique sociocultural interspecies dynamic and practical strategies for improving the wellbeing of both the humans and the dairy bovines of India.

The research expanded the validity of the TPB model to a non-Western context, specifically with relation to the model’s suitability to measure animal-based food consumption intentions. The results demonstrate the strong influence each of the three TPB variables exerts on behavioral intentions, with subjective norms being the most significant in shaping behavioral intentions. Furthermore, the study makes a substantial theoretical contribution by looking at religious beliefs as a moderating element. The manner in which cow-related religious beliefs moderate the relationships between behavioral intention and subjective norms has not been explored in previous research. The main conclusions of this study, however, reveal that these religious beliefs have an amplifying effect on, and in fact exhibit, an association with these relationships. Thus, cow-related religious beliefs exhibit the ability to mold the subjective norms around the consumption of cow dairy products among Indians. This warrants further research into this construct’s ability to shape other consumption and non-consumption-related subjective norms around the cow and its broader applicability as a distinct moderating construct in the TPB model.

In terms of practical implications, this study sets the groundwork for policy development and program design to improve the wellbeing of the Indian dairy bovines by redefining and rebuilding Indians’ approach towards the revered cow. The research pioneers an insight into the psyche of the common end customer of the Indian dairy business, whose dairy consumption, without their knowledge, can be responsible for compromising the welfare of the same animal whom they revere. This conceptual paradox, therefore, calls for public awareness generation efforts regarding the effects of Indian dairy farming practices and the religiously originated cow slaughter ban on cows’ life quality. It also warrants the re-animalization and re-individualization of the de-animalized, religiously commodified cow through formal and informal education channels that utilize the animal’s biological, mammalian proximity to human beings to communicate her animality, her femininity, her motherhood, and her personhood. It appeals for prohibiting the religious emblematization of cow milk and milk products, which seek to promote the sale of the dairy products by abusing Indians’ cow-related religious beliefs. Instead, it necessitates an appropriate use of these religious beliefs to improve the cows’ wellbeing by diverting adequate monetary funds to employ scientifically sound methods of treating the dairy animal humanely before, during, *and* after her productive phase. Creation of such laws and regulations, at both the national and state levels, can foster the Indian dairy industry’s compliance with these initiatives and help Indians rethink their choices by evaluating the effects of their culturally originated behaviors on the animal they revere.

Future research should examine Indians’ awareness of the cows’ animality, and of the implications of the cow’s religious commodification on the animal’s wellbeing. Research should also address the influences of modernization, including technological advancements and urbanization, which reduce human exposure to ubiquitous small-scale suburban and rural dairy farms, provide easy access to packaged milk and milk products, and thereby draw a curtain between the cow-worshiping consumer and the living, animate source of the milk and milk products—the Indian dairy cow. Further, scholarship should analyze how the non-cow-deifying marginalized communities of India view the animal and, therefore, how they treat the animal and the food derived from her. Exploring such sparsely explored research areas can create the foundation for the generation of culturally sustainable human–bovine relationships, which promote the wellbeing of both the humans and the bovines of India.

## Figures and Tables

**Table 1:** Research variables, corresponding measurement scales, and descriptive statistics.

Variable		Item	Mean ± SD
Behavioral Intentions (α = 0.85)	1	How likely are you to consume cow milk or milk products regularly during the next month?	4.14 ± 0.83
	2	How often will you consume cow milk or milk products during the next month?	4.18 ± 1.28
	1	Consuming cow milk or milk products is extremely bad—extremely good	4.43 ± 0.66
Attitudes (α = 0.79)	2	Consuming cow milk or milk products is extremely harmful—extremely beneficial	3.98 ± 0.70
	3	Consuming cow milk or milk products is extremely unenjoyable—extremely enjoyable	3.56 ± 1.03
	1	Do the people who are important to you (family, friends, colleagues) think that you should consume cow milk or milk products regularly?	4.60 ± 0.85
Subjective norms (α = 0.79)	2	Do your health care providers (doctor, nutritionist) think that you should consume cow milk or milk products regularly?	4.37 ± 0.51
	3	Do you think that consuming cow milk or milk products regularly sets a good example for your community members?	3.97 ± 0.70
	1	Can you decide for yourself whether or not to consume cow milk or milk products?	4.28 ± 0.68
Perceived behavioral control (α = 0.82)	2	Can you decide for yourself when or when not to consume cow milk or milk products?	4.10 ± 1.09
	3	How easily are cow milk or milk products accessible to you?	4.53 ± 1.05
Religious beliefs	1	Do you consider the cow sacred?	4.05 ± 1.26

## Data Availability

The data created in this study are available on request from the corresponding author.

## Abbreviations

The following abbreviation is used in this manuscript:

TPB	Theory of Planned Behavior
